# Study on the Characteristics of Traditional Chinese Medicine Syndromes in Patients with Erosive Gastritis Based on Metabolomics

**DOI:** 10.1155/2024/6684677

**Published:** 2024-01-02

**Authors:** Zhang Shixiong, Liu Shaowei, Yang Zeqi, Xu Miaochan, Zhou Pingping, Bai Haiyan, Lv Jingjing, Wang Yangang

**Affiliations:** ^1^Nanjing University of Chinese Medicine, Nanjing 210046, China; ^2^Hebei University of Chinese Medicine, Shijiazhuang 050200, China; ^3^Hebei Hospital of Traditional Chinese Medicine, Shijiazhuang 050091, China; ^4^The Third Affiliated Hospital of Beijing University of Chinese Medicine, Beijing 100029, China

## Abstract

According to traditional Chinese medicine theory, tongue coatings reflect changes in the body. The goal of this study was to identify a metabolite or a set of metabolites capable of classifying characteristics of traditional Chinese medicine syndromes in erosive gastritis. In this study, we collected tongue coatings of patients with erosive gastritis with damp-heat syndrome (DHS), liver depression and qi stagnation syndrome (LDQSS), and healthy volunteers. Then, we analyzed the differences in metabolic characteristics between the two groups based on metabolomics. We identified 14 potential biomarkers related to the DHS group, and six metabolic pathways were enriched. The differential pathways included pyrimidine metabolism, pantothenate and CoA biosynthesis, citrate cycle (TCA cycle), pyruvate metabolism, glycolysis/gluconeogenesis, and purine metabolism. Similarly, in the LDQSS group, we identified 25 potential biomarkers and 18 metabolic pathways were enriched. The top five pathways were the TCA cycle, sphingolipid metabolism, fatty acid biosynthesis, pantothenate and CoA biosynthesis, and the pentose phosphate pathway. In conclusion, the DHS group and the LDQSS group have different characteristics.

## 1. Introduction

Erosive gastritis (EG) is one of the most common diseases in the world [1]. Currently, the main diagnostic method for EG is electronic gastroscopy [2]. However, this method is often too invasive for patients to tolerate [3]. EG causes great trouble, although it generally does not endanger the life of patients. Therefore, it is worth exploring a simple and noninvasive diagnostic method to identify the characteristics of EG.

According to traditional Chinese medicine theory, tongue coatings reflect changes in the body [4, 5]. Based on this theory, it has been found that tongue features can be used as a stable method for disease diagnosis [6]. Tongue coating evaluation is sometimes even significantly superior to the use of traditional blood biomarkers [7]. In accordance with this theory, EG can be classified into two distinct syndromes: damp-heat syndrome (DHS) and liver depression and qi stagnation syndrome (LDQSS) [8]. Based on the different EG syndromes, traditional Chinese medicine (TCM) can provide more effective personalized treatment according to the features of each syndrome [9]. While previous studies have focused on bacteria from different tongue coatings in EG, variations in their metabolites remain unknown.

As a sensitive indicator for judging the condition of EG, tongue coatings play a pivotal role in the effective diagnosis and treatment [10]. Due to the comprehensive, highly sensitive, specific, and noninvasive nature of metabolomics, it has recently become a new research focus to promote disease diagnosis and is thought to be the most predictive phenotype [11]. In recent years, metabolomics has widely been used in the research of tongue coatings, such as in gastric precancerous lesions, coronary heart disease, and chronic renal failure [12, 13], with a particular focus on chronic gastritis [10]. We have identified changes in the salivary metabolome in patients with EG in our previous study [14]. We carried out the present study to further explore the metabolomics changes in EG patients. Herein, we collected tongue coatings of patients with EG with DHS and LDQSS, the two most common conditions in TCM. Due to their prominence in clinical practice, these two conditions have been listed in the International Classification of Diseases by the World Health Organization [15]. However, their biological characteristics have not yet been systematically studied. Therefore, in this study, we used nontargeted metabolomics methods to study the tongue coating samples of EG patients with DHS and LDQSS and to screen the metabolic products with characteristic significance.

## 2. Materials and Methods

### 2.1. Patients

**Figure 1:**
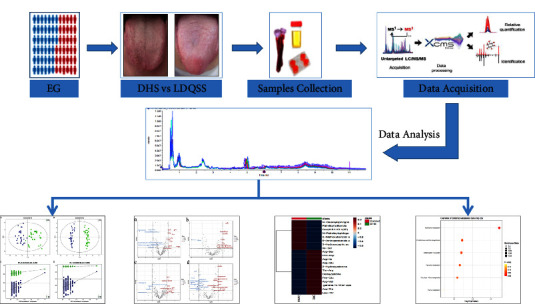
Experimental procedure.

We conducted a case control study to elucidate the composition of tongue coating-related metabolites in EG patients with DHS and LDQSS. A total of 64 patients with EG were selected from the Hebei Provincial Hospital of Traditional Chinese Medicine, including 32 cases with DHS and 32 cases with LDQSS. In addition, we selected 30 healthy volunteers, all of whom were either college students or hospital staff who did not have any digestive system symptoms and who passed the physical examination, tongue coating evaluation, and pulse condition assessment at the Hebei Province Hospital of Traditional Chinese Medicine. The experimental procedure is shown in Figure 1.

### 2.2. Ethics Approval

All of the subjects signed a written informed consent form before sample collection, and the study was conducted in accordance with the Declaration of Helsinki. In addition, the study was approved by the Ethics Committee of Hebei Province Hospital of Traditional Chinese Medicine (HBZY2021-KY-045-01).

### 2.3. Diagnostic Criteria of EG

#### 2.3.1. Diagnostic Criteria in Gastroscopy

According to the diagnostic criteria for EG formulated by the Digestive Branch of the Chinese Medical Association in 2017, the diagnosis of EG includes single or multiple erosive lesions in the gastric mucosa that range in size from the size of a needle tip to several centimeters, or else single or multiple verruciform, bulging folds or papuloid protrusions in the gastric mucosa with diameters of 5–10 mm, a mucosal defect or umbilical-like depression at the top, and erosion at the center [16].

#### 2.3.2. Diagnostic Criteria in Pathology

According to the pathological diagnostic criteria in the consensus on the Diagnosis and Treatment of Chronic Gastritis by Integrated Traditional Chinese and Western Medicine, mucosal infiltration with monocytes or neutrophils is the main manifestation of EG [8].

#### 2.3.3. DHS and LDQSS Diagnostic Criteria

According to the TCM syndrome standard in the consensus on the Diagnosis and Treatment of Chronic Gastritis by Integrated Traditional Chinese and Western Medicine [8]:

DHS is defined as follows: having two main symptoms + one secondary disease, or one main symptom + two secondary diseases, combined with an evaluation based on gastroscopic findings.

The main symptoms are ① bloating or stomachache and ② red tongue with yellow, greasy, or thick fur.

Minor symptoms include ① heartburn in the stomach; ② bitter mouth and bad breath; ③ nausea and vomiting; ④ sticky stool; and ⑤ a slippery or wet pulse.

Relevant gastric results include ① thick and turbid mucus and ② obviously congested, edematous, and erosive gastric mucosa.

LDQSS is defined as follows: having two main symptoms + one secondary symptom, or the first main symptom + two secondary symptoms, combined with an evaluation based on gastroscopic findings.

The main symptoms include ① epigastric distension or pain in both flanks; ② pain due to emotional factors; and ③ pulse string.

Minor symptoms include ① frequent belching; ② chest tightness or excessive breathing; ③ lack of appetite; ④ mental depression; and ⑤ a light red tongue with thin white fur; relevant gastric results include ① active or slow peristalsis; ② erythema of the gastric mucosa, in the form of dots, patches, or strips; and ③ bile reflux.

**Figure 2:**
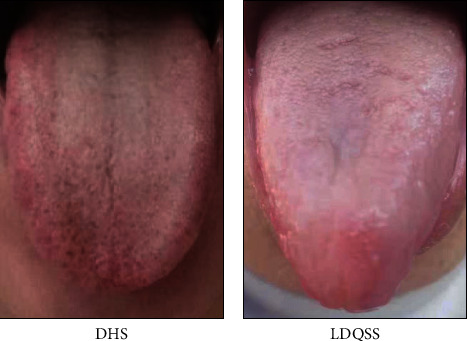
Typical photographs taken from the patients with (a) DHS and (b) LDQSS.

Typical photographs taken from the patients who participated in the research are presented in Figure 2.

### 2.4. Sample Collection

#### 2.4.1. Tongue Collection

First, we asked the subjects to clean their oral cavity for three times with distilled water free of any impurities. Then, we gently scraped an appropriate amount of tongue coating on the surface of the participant's tongue. Finally, the scraped samples were put into a centrifuge tube and immediately stored at −80°C until further analysis [17].

#### 2.4.2. Sample Extraction

The samples were thawed without any damage. Then, 20 mg of the samples were taken, and 50 *μ*l of water and 200 *μ*l of methanol/acetonitrile solution (1 : 1, v/v) were added before vortex mixing. After that, the samples were sonicated at low temperature for 30 min. Then, they were centrifuged for 20 minutes at 14000 g at 4°C. The supernatant was collected, and 5 *μ*l was injected into the instrument for final detection [18].

#### 2.4.3. UHPLC-Q/TOF-MS Conditions

The samples were separated using an Agilent 1290 Infinity LC Ultra Performance Liquid Chromatography System HILIC column. The AB Triple TOF 6600 mass spectrometer was used to collect the primary and secondary spectra of the samples [19–21].

For HILIC separation, samples were analyzed using a 2.1 mm × 100 mm ACQUIY UPLC BEH 1.7 *µ*m column (waters, Ireland). In both ESI positive and negative modes, the mobile phase contained *A* = 25 mM ammonium acetate and 25 mM ammonium hydroxide in water and *B* = acetonitrile. The gradient was 85% *B* for 1 min and was linearly reduced to 65% in 11 min and then was reduced to 40% in 0.1 min and kept for 4 min and then increased to 85% in 0.1 min, with a 5 min re-equilibration period employed.

For RPLC separation, a 2.1 mm × 100 mm ACQUIY UPLC HSS T3 1.8 *µ*m column (waters, Ireland) was used. In ESI positive mode, the mobile phase contained *A* (water with 0.1% formic acid) and *B* (acetonitrile with 0.1% formic acid); and in ESI negative mode, the mobile phase contained *A* (0.5 mM ammonium fluoride in water) and *B* (acetonitrile). The gradient was 1% *B* for 1.5 min and was linearly increased to 99% in 11.5 min and kept for 3.5 min. Then, it was reduced to 1% in 0.1 min, and 3.4 min of the re-equilibration period was employed. The gradients were at a flow rate of 0.3 mL/min, and the column temperatures were kept constant at 25°C.

The ESI source conditions were set as follows: Ion Source Gas1 (Gas1) was 60 psi, Ion Source Gas2 (Gas2) was 60 psi, curtain gas (CUR) was 30 psi, source temperature was 600°C, and IonSpray Voltage Floating (ISVF) was ±5500 V. In MS only acquisition, the instrument was set to acquire over the m/z range 60–1000 Da, and the accumulation time for TOF MS scan was set at 0.20 s/spectra. In auto MS/MS acquisition, the instrument was set to acquire over the m/z range 25–1000 Da, and the accumulation time for product ion scan was set at 0.05 s/spectra. The product ion scan was acquired using information dependent acquisition (IDA) with a high sensitivity mode selected. The collision energy (CE) was fixed at 35 V with ±15 eV; declustering potential (DP) was 60 V (+) and −60 V (−); exclude isotopes were set within 4 Da, and candidate ions to monitor per cycle were 10.

### 2.5. Statistical Analysis

A chi-square test was used for intergroup comparisons of gender, and the Mann–Whitney *U* test was used for intergroup comparison of age. This study relied on MetaboAnalyst 5.0 (https://www.metaboanalyst.ca) to find significant differences between the groups. Data analysis was mainly based on fold-change analysis (FC Analysis), variable importance for the projection (VIP), and orthogonal partial least squares discrimination analysis (OPLS-DA) [22].

## 3. Results

### 3.1. Basic Information

A total of 32 EG patients with DHS (15 males and 17 females) and 30 healthy volunteers (15 males and 15 females) were included. There were no intergroup differences in gender distribution (*P* > 0.05), and age did not differ significantly between the DHS group with average 49.63 ± 9.76 years and the control group with average 50.13 ± 8.79 years (*P* > 0.05). We also included 32 EG patients with LDQSS (15 males and 17 females) and the 30 healthy volunteers mentioned above. There were no intergroup differences in gender distribution (*P* > 0.05), and age did not differ significantly between the LDQSS group with average 49.81 ± 9.56 years and the control group with average 50.13 ± 8.79 years (*P* > 0.05). The above data can be found in the supplementary material (available here).

### 3.2. OPLS-DA

**Figure 3:**
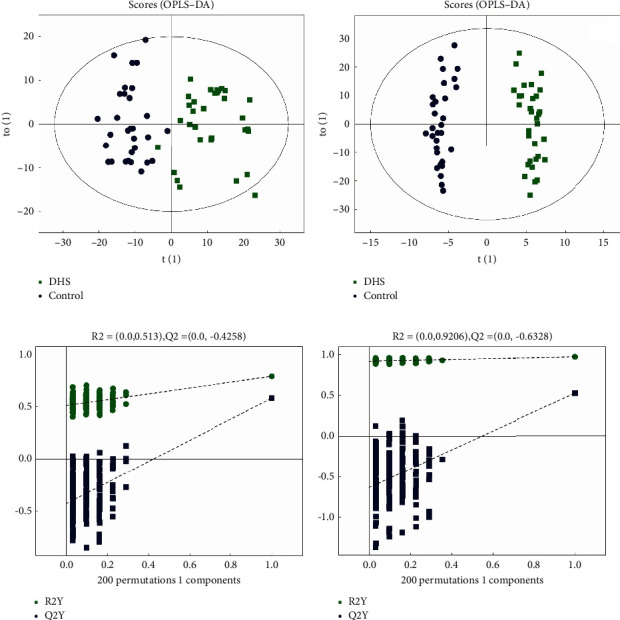
OPLS-DA analysis of metabolomics data between the DHS group and the control group. (a) Comparison between the two groups in the positive ion mode. (b) Permutation test results in the positive ion mode. (c) Comparison between the two groups in the negative ion mode. (d) Permutation test results in the negative ion mode. The DHS group is represented by green dots, and the control group is represented by blue dots.

**Figure 4:**
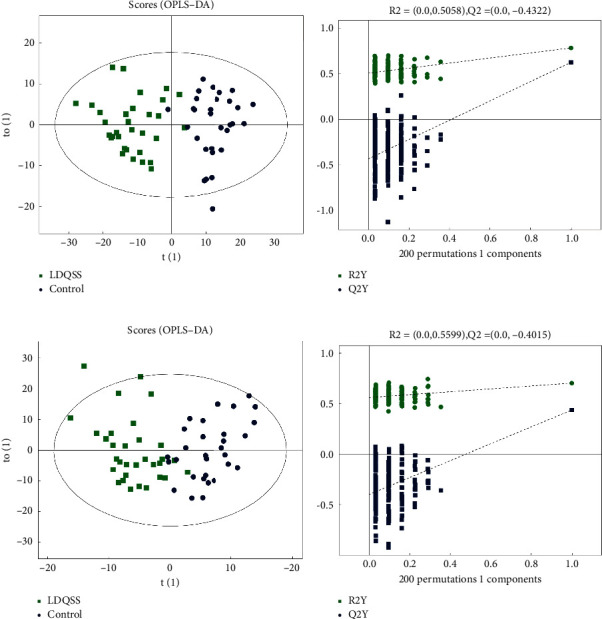
OPLS-DA analysis of metabolomics data between the LDQSS group and the control group. (a) Comparison between the two groups in the positive ion mode. (b) Permutation test results in the positive ion mode. (c) Comparison between the two groups in the negative ion mode. (d) Permutation test results in the negative ion mode. The LDQSS group is represented by green dots, and the control group is represented by blue dots.

There were 481 metabolites by identification, 313 for positive and 229 for negative; they were used to generate the OPLS-DA models. In this study, OPLS-DA was performed between the DHS group and the control group based on the positive and negative ion modes. To avoid overfitting of the supervised model in the modeling process, we used the permutation test to check the model. The results showed that the two groups were well distinguished (Figure 3), and the model had never been fitted. OPLS-DA was also performed between the LDQSS group and the control group. The results showed that the two groups were also well distinguished (Figure 4), and the model had never been fitted.

### 3.3. Volcano Plot

**Figure 5:**
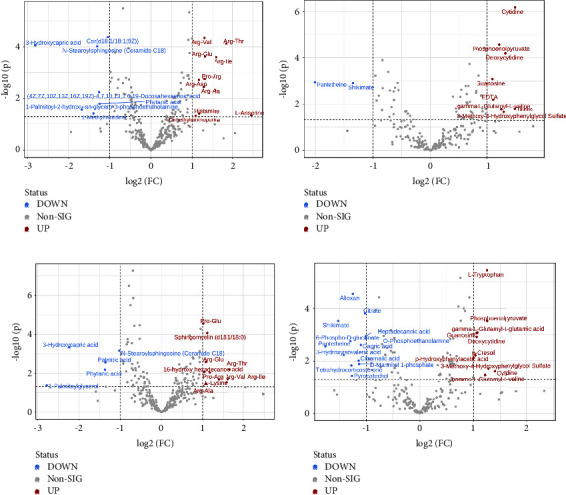
Results of volcano plot. (a) Between the DHS group and the control group in the positive ion mode. (b) Between the DHS group and the control group in the negative ion mode. (c) Between the LDQSS group and the control group in the positive ion mode. (d) Between the LDQSS group and the control group in the positive ion mode.

Based on univariate analysis, the differential metabolites between the DHS group and the control group were determined. We analyzed all metabolites detected in positive and negative ion modes. The differential metabolites with a FC > 2 or FC < 0.5 and a *P* value <0.05 were visualized in the form of a volcano plot. The differential metabolites between the LDQSS group and the control group were also analyzed. The results are shown in Figure 5.

### 3.4. Differential Metabolites

In this study, strict thresholds of VIP > 1 and *P* value <0.05 were used as the screening criteria for metabolites with significant differences between the DHS group and the control group. We demonstrated differential metabolites with FC > 2 or FC < 0.5. The results are shown in Table 1. The metabolites with significant differences between the LDQSS group and the control group were determined. The results are shown in Table 2.

### 3.5. Cluster Analysis

**Figure 6:**
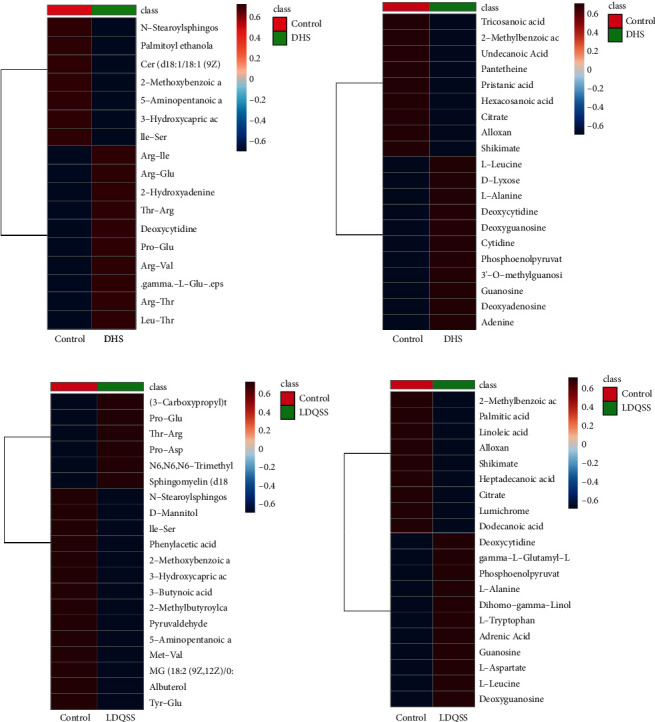
Results of cluster analysis. (a) Between the DHS group and the control group in the positive ion mode. (b) Between the DHS group and the control group in the negative ion mode. (c) Between the LDQSS group and the control group in the positive ion mode. (d) Between the LDQSS group and the control group in the positive ion mode.

To more comprehensively and intuitively display the differences, we conducted cluster analysis on the above results. Metabolites in the same cluster have similar expression patterns and may have similar functions or participate in the same metabolic process or cellular pathway together. Heat maps were generated to visualize the altered pattern of the significantly different metabolites (Figure 6).

### 3.6. Biomarker Analysis

**Figure 7:**
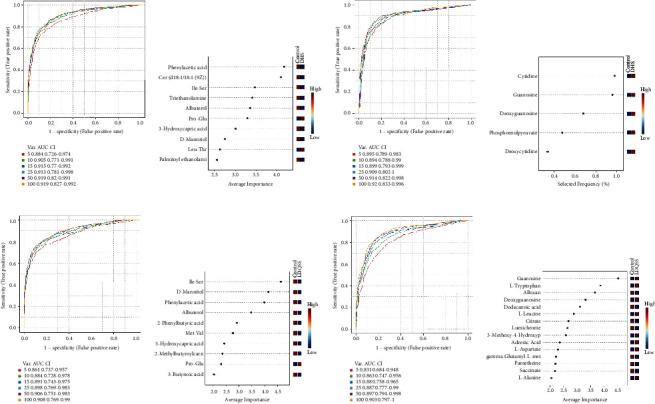
Results of ROC curve analysis. (a) Between the DHS group and the control group in the positive ion mode. (b) Plot of the most important features of a selected model ranked from most to least important between the DHS group and the control group in the positive ion mode. (c) Between the DHS group and the control group in the negative ion mode. (d) Plot of the most important features of a selected model ranked from most to least important between the DHS group and the control group in the negative ion mode. (e) Between the LDQSS group and the control group in the positive ion mode. (f) Plot of the most important features of a selected model ranked from most to least important between the LDQSS group and the control group in the positive ion mode. (g) Between the LDQSS group and the control group in the negative ion mode. (h) Plot of the most important features of a selected model ranked from most to least important between the LDQSS group and the control group in the negative ion mode.

Biomarker discovery was achieved through building predictive models of multiple metabolites to classify the patients into different categories. In this study, we chose random forest (RF) as the multivariate algorithm for ROC curve analysis. ROC curves for biomarkers between the DHS group and the control group were plotted based on the average performance runs (Figures 7(a)–7(c)), and the significant features of the biomarker model were ranked by importance (Figures 7(b)–7(d)). Likewise, ROC curves for biomarkers between the LDQSS group and the control group were plotted based on the average performance runs (Figures 7(e)–7(g)), and the significant features of the biomarker model were ranked by importance (Figures 7(f)–7(h)).

### 3.7. KEGG Analysis

**Figure 8:**
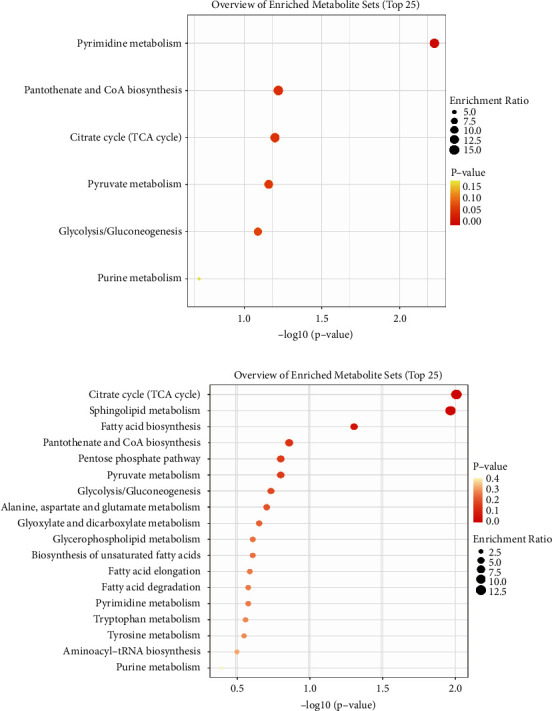
Results of KEGG analysis. (a) Between the DHS group and the control group. (b) Between the LDQSS group and the control group.

On the basis of the previous work, we conducted enrichment analysis on the selected significantly different metabolites. The results showed that pyrimidine metabolism, pantothenate and CoA biosynthesis, citrate cycle (TCA cycle), pyruvate metabolism, glycolysis/gluconeogenesis, and purine metabolism played an important role in the comparison between the DHS group and the control group (Figure 8(a)). Of note, the LDQSS group involved more pathways, and the top five were TCA cycle, sphingolipid metabolism, fatty acid biosynthesis, pantothenate and CoA biosynthesis, and pentose phosphate pathway (Figure 8(b)).

## 4. Discussion

Due to the comprehensive, highly sensitive, specific, and noninvasive nature of metabolomics, it has become a new research focus in disease diagnosis research in recent years. As one of the main methods for finding potential diagnostic biomarkers, metabolomics has extensively been used in the research on tongue coating [23]. There is mounting evidence that metabolic changes are associated with the initiation and development of tongue coatings [10, 12, 13]. Previous studies have demonstrated that gastric precancerous lesions, coronary heart disease, and chronic renal failure [12, 13], but especially chronic gastritis [10], have unique metabolic characteristics. However, although EG is a common type of chronic gastritis, the tongue metabolic profile of EG has not been thoroughly studied, and the underlying mechanism of different syndromes of EG remains unknown. Therefore, we carried out the present study based on metabolomics to discriminate between different features to improve diagnosis.

In this study, the baseline data did not significantly deviate between the DHS group and the control group. The results showed that the differences between the two were not affected by factors such as age and gender. In the comparison between the DHS group and the control group, the OPLS-DA results showed a significant difference between the two groups. The results proved that there was clear differentiation between the two groups. The volcano plot analyses also showed alterations in various metabolites; some were elevated, while others were decreased. In order to obtain more comprehensive and accurate results, characteristic metabolites were detected simultaneously in both positive and negative ion modes. In both modes, different metabolites are detected, but sometimes, there may be duplication. In the positive mode, there were 10 metabolites with an increasing trend. Among them, L-anserine, a bioactive dipeptide found in muscles and brains of vertebrates, was the most elevated metabolite [24]. There were seven metabolites with a downward trend. Among them, 3-hydroxycapric acid was the most significantly reduced metabolite. Along with L-anserine, they were enriched as potential spoilage biomarkers [25], consistent with erosion in the EG. In the negative mode, there were eight metabolites with an increasing trend. Of them, cytidine was the most significantly changed. There were two metabolites with a downward trend. Among them, pantetheine acid was the most significantly changed. Both of them have been used as potential biomarkers for unclassified patients with pediatric-onset multiple sclerosis [26]. The discovery of them may serve as an important potential marker for DHS.

The common goal of this study was to identify a metabolite or a set of metabolites capable of classifying characteristics of TCM syndromes in EG with high sensitivity (true positive rate) and specificity (true negative rate). So, we identified metabolites with significant differences that could act as biomarkers for distinguishing between the two groups by ROC curve analysis. The results showed that these were phenylacetic acid, Cer (d18 : 1/18 : 1(9Z)), Ile-Ser, triethanolamine, albuterol, Pro-Glu, 3-hydroxycapric acid, D-mannitol, Leu-Thr, palmitoyl ethanolamide in the positive mode, and cytidine, guanosine, deoxyguanosine, phosphoenolpyruvate, and deoxycytidine in the negative mode. We also conducted cluster analysis to enhance the reliability of the results. Based on the enrichment analysis, it was found that pyrimidine metabolism, pantothenate and CoA biosynthesis, TCA cycle, pyruvate metabolism, glycolysis/gluconeogenesis, and purine metabolism played an important role in the comparison between the DHS group and the control group. Pyrimidine metabolism, glycolysis/gluconeogenesis, and purine metabolism are closely linked to inflammation and oxidative stress, the commonly accepted mechanistic pathway associated with marked susceptibility to infection [27–29]. CoA in the pathway of pantothenate and CoA biosynthesis is mainly involved in the metabolism of pyruvate, which can stimulate TCA cycle and provide 90% of the energy requirements for the body. This result indicated that there were differences in energy changes in the DHS group [30].

Similarly, in the comparison between the LDQSS group and the control group, the same operation was performed and satisfactory results were also obtained. OPLS-DA results also showed a significant difference between the two groups, proving that the two groups have significant heterogeneity. The volcano plot results showed up or downregulated differential metabolites. Interestingly, the different metabolites between LDQSS and DHS were the same in the negative mode, while they were different in the positive mode; they were Arg-Thr and 1-palmitoylglycol, respectively. Next, we identified the metabolites with significant differences, which can act as biomarkers for distinguishing between the two groups. The results of ROC curve analysis showed that they were Ile-Ser, D-mannitol, phenylacetic acid, albuterol, 2-phenylbutyric acid, Met-Val, 3-hydroxycapric acid, 2-methylbutyroylcarnitine, Pro-Glu, and 3-butynoic acid in the positive mode, and guanosine, L-tryptophan, alloxan, deoxyguanosine, dodecanoic acid, L-leucine, citrate, lumichrome, 3-methoxy-4-hydroxyphenylglycol sulfate, adrenic acid, L-aspartate, gamma-glutamyl-L-methionine, pantetheine, succinate, and L-alanine in the negative mode. We also conducted cluster analysis to enhance the reliability of the results. Based on the enrichment analysis, it was found that the LDQSS group involved more metabolic pathways, and the top five were TCA cycle, sphingolipid metabolism, fatty acid biosynthesis, pantothenate and CoA biosynthesis, and pentose phosphate pathway. Among them, the TCA cycle, fatty acid biosynthesis, pantothenate and CoA biosynthesis, and pentose phosphate pathways were largely similar to the DHS group. Moreover, the function of sphingolipid metabolism is immense and touches almost all major aspects of cell biology, including roles in cell growth, cell cycle, cell death, cell senescence, inflammation, immune responses, cell adhesion and migration, angiogenesis, nutrient uptake, metabolism, responses to stress stimuli, and autophagy [31].

The abovementioned results fully demonstrated the differences in metabolic characteristics between the LDQSS group and the DHS group. These findings not only provide technical support for the tongue for EG diagnosis but also reflect that different signs of EG have different characteristics, thereby providing support for further precision treatment and research basis for further exploration of the potential mechanisms of different signs based on metabolomics. In the future, the metabolites identified in this study may be used as noninvasive and convenient biomarkers to distinguish DHS and LDQSS of EG patients.

## 5. Conclusions

Taken together, this study revealed that EG with DHS and EG with LDQSS have different characteristics. In summary, 14 potential biomarkers related to the DHS group were identified and six metabolic pathways were enriched. The differential metabolites were enriched in pyrimidine metabolism, pantothenate and CoA biosynthesis, TCA cycle, pyruvate metabolism, glycolysis/gluconeogenesis, and purine metabolism, pathways related to inflammation, oxidative stress, and energy change. Similarly, in the LDQSS group, 25 potential biomarkers were identified and 18 metabolic pathways were enriched. The top five pathways were TCA cycle, sphingolipid metabolism, fatty acid biosynthesis, pantothenate and CoA biosynthesis, and pentose phosphate pathway. The results showed that the LDQSS group involved more metabolic pathways than the DHS group. There was consistency in the metabolic pathways involved between the two groups, but there were also significantly different pathways. Among them, the function of sphingolipid metabolism is immense and touches almost all major aspects of cell biology. Based on the above results, we hope that this research can provide reference and guidance for follow-up research and even lay the foundation for its application in TCM clinical diagnosis and treatment. However, there are still many shortcomings in this research. We hope that related research can be carried out in the future.

## Figures and Tables

**Table 1:** Differential metabolites in the positive ion mode.

Mode	Names	VIP value	FC	*P* value
POS	Cer (d18 : 1/18 : 1(9Z))	2.3520	0.4844	0.00
POS	Arg-Val	2.3244	2.4564	0.00
POS	Arg-Thr	2.2625	3.6149	0.00
POS	3-Hydroxycapric acid	1.9568	0.1426	0.00
POS	N-Stearoylsphingosine (ceramide C18)	2.2985	0.4047	0.00
POS	Arg-Glu	2.0946	2.4844	0.00
POS	Arg-Ile	2.1279	3.0491	0.00
POS	Pro-Arg	1.9208	2.4638	0.00
POS	Arg-Asp	1.7709	2.2461	0.00
POS	Arg-Ala	1.7586	2.4340	0.00
NEG	Cytidine	2.6703	2.7989	0.00
NEG	Phosphoenolpyruvate	2.2585	2.3120	0.00
NEG	Deoxycytidine	2.3083	2.4908	0.00
NEG	Guanosine	1.8122	2.1315	0.00
NEG	Pantetheine	1.7972	0.2491	0.00
NEG	Shikimate	1.8609	0.3931	0.00
NEG	EDTA	1.4562	2.1516	0.01

**Table 2:** Differential metabolites in the negative ion mode.

Mode	Names	VIP value	FC	*P* value
POS	Pro-Glu	2.1049	2.0158	0.00
POS	Sphingomyelin (d18 : 1/18 : 0)	2.0866	2.1614	0.00
POS	3-Hydroxycapric acid	1.6420	0.2131	0.00
POS	N-Stearoylsphingosine (ceramide C18)	1.9000	0.4935	0.00
POS	Arg-Glu	1.8303	2.1123	0.00
POS	Palmitic acid	1.4477	0.3911	0.00
NEG	L-Tryptophan	2.2527	2.3806	0.00
NEG	Alloxan	2.1289	0.4201	0.00
NEG	Citrate	2.0791	0.4924	0.00
NEG	Phosphoenolpyruvate	1.6457	2.3873	0.00
NEG	Shikimate	1.9389	0.3477	0.00
NEG	Gamma-L-glutamyl-L-glutamic acid	1.7349	2.1103	0.00
NEG	Guanosine	1.6253	2.0845	0.00
NEG	Heptadecanoic acid	1.5218	0.4978	0.00
NEG	Deoxycytidine	1.4530	2.0842	0.00
NEG	O-Phosphoethanolamine	1.6739	0.4994	0.00
NEG	6-Phospho-D-gluconate	1.7636	0.4074	0.00
NEG	Capric acid	1.3734	0.4662	0.00
NEG	Pantetheine	1.6168	0.2956	0.00
NEG	3-Hydroxyisovaleric acid	1.3577	0.3742	0.01
NEG	P-Cresol	1.3360	2.0519	0.01
NEG	p-Hydroxyphenylacetic acid	1.2972	2.0004	0.01
NEG	Citramalic acid	1.4332	0.4531	0.01

## Data Availability

The omics data used to support the findings of this study are available from the corresponding author upon request.

## Supplementary Materials

Supplementary Materials The information of gender and age.
