# Supplementary material for: Study on the Characteristics of Traditional Chinese Medicine Syndromes in Patients with Erosive Gastritis Based on Metabolomics
Source: Int J Anal Chem. 2024 Jan 2;2024:6684677. doi: 10.1155/2024/6684677 (PMC10776191; doi:10.1155/2024/6684677)
Supplement: Supplementary Materials — The information of gender and age. [file 6684677.f1.pdf]

Gender	DHS	LDQSS	Volunteers(Control)
male	15	15	15
female	17	17	15

Groups

DHS vs Control	Group	Gender	Number
	1	1	15
	1	2	17
	2	1	15
	2	2	15
x^2	P value		
0.061	0.806		

Groups

LDQSS vs Control	Group	Gender	Number
	1	1	15
	1	2	17
	2	1	15
	2	2	15
x^2	P value		
0.061	0.806		

	DHS	LDQSS	Volunteers(Control)
1	47	52	42
2	51	52	47
3	56	59	50
4	28	69	50
5	60	48	49
6	50	65	30
7	57	54	51
8	46	51	60
9	53	54	53
10	29	52	48
11	48	38	50
12	46	52	32
13	58	39	46
14	52	45	44
15	24	53	49
16	41	35	52
17	45	62	57
18	44	54	50
19	61	39	57
20	56	56	36
21	60	52	68
22	58	44	57
23	59	43	58
24	62	63	56
25	56	56	62
26	39	46	40
27	40	37	62
28	49	25	49
29	52	41	56
30	59	55	43
31	54	46	
32	48	57	
Mean	49.63	49.81	50.13
Standard Deviatio	9.764	9.563	8.729

Groups	*P* value
DHS vs Control	0.155
LDQSS vs Control	0.432
